# The Ideas Lab Concept, Assembling the Tree of Life, and AVAToL

**DOI:** 10.1371/currents.tol.0fdb85e1619f313a2a5a2ec3d7a8df9e

**Published:** 2013-03-07

**Authors:** Timothy Collins, Maureen Kearney, David Maddison

**Affiliations:** Florida International University; National Science Foundation; Oregon State University

## Abstract

In August 2011, a week-long NSF-sponsored workshop focusing on the Tree of Life (ToL) took place in Lake Placid, New York. This workshop, called AVAToL (Assembling Visualizing, and Analyzing the Tree of Life), was the first application of NSF’s Ideas Lab concept to systematics. In this article we outline the history and motivation for the Ideas Lab approach and its application to the ToL, explain the nuts and bolts of the Ideas Lab process and look to the potential contributions of AVAToL funded projects to help enable the future of ToL and more broadly, comparative biological research.

## Introduction

Tens of millions of species inhabit Earth, each with its own complex history. The core structure of this history, formed during billions of years of evolution, is the Tree of Life (ToL). The ToL unifies biology by providing an evolutionary framework for interpretation of all comparative information about organisms. It is now recognized that comparative biological studies must account for phylogenetic relatedness because all organisms’ interactions and processes are, to some extent, shaped and/or constrained by their phylogenetic history 1. With knowledge of phylogenetic trees, biologists investigate patterns and processes of trait evolution, connect phylogenetic patterns with geographic distributions through time, link evolutionary history to patterns of ecosystem assembly, connect genomes to phenomes, and achieve a greater overall understanding of biodiversity.

In August 2011, a week-long NSF-sponsored workshop focusing on the ToL took place in Lake Placid, New York. This workshop, called AVAToL (Assembling Visualizing, and Analyzing the Tree of Life), was the first application of NSF’s Ideas Lab concept to systematics. In this article we outline the history and motivation for the Ideas Lab approach and its application to the ToL, explain the nuts and bolts of the Ideas Lab process, and look to the potential contributions of AVAToL funded projects to help enable the future of ToL and, more broadly, comparative biological research.

## History of the Ideas Lab Concept and the Ideas Lab Process

What is an Ideas Lab, and how does it differ from a typical NSF workshop or solicitation? Ideas Labs are intensive, interactive, five-day residential workshops where 20-30 participants come together to develop novel and potentially transformative approaches to a grand challenge. A major difference with respect to typical NSF solicitations is that new multidisciplinary teams are formed *during* the Ideas Lab, rather than *before* proposal submission. These new multidisciplinary teams form around novel ideas, which are then refined through an iterative development and feedback process that involves both team members and other participants in the Ideas Lab. Ideas Labs differ from standard NSF exploratory workshops in that specific funding is associated with the lab, and may be awarded following the Ideas Lab to successful teams and ideas.

The Ideas Lab concept originated with the United Kingdom’s Engineering and Physical Sciences Research Council (EPSRC), where they are called “Sandpits” (http://www.epsrc.ac.uk/funding/grants/network/ideas/Pages/experience.aspx). The Sandpit, and more broadly the “IDEAS Factory”, were developed in response to the perception of disciplinary “siloing” or “stove-piping” — that is, many mature research communities are settled within field-specific paradigms or research programs, while transformative research seemed more often to be the result of multidisciplinary/integrative approaches. The first Sandpit, on angiogenesis, was held in Dundee Scotland in December of 2003 2 Since that time, Sandpits have become a routine component of EPSRC’s funding strategy. A search of EPSRC’s Grants on the Web site (http://gow.epsrc.ac.uk/Search.aspx) using the term “sandpit” in October 2012 yielded 363 hits on topics such as The Programmable Rhizosphere, Transgenerational Effects and Evolution, Evolving Robotic Culture, SerenA - Chance Encounters in the Space of Ideas, and Integrating and Automating Airport Operations. Overall, the EPSRC experience is that sandpits are producing results that are markedly different from those funded through the typical grants process (http://www.epsrc.ac.uk/SiteCollectionDocuments/Publications/corporate/IDEASBrochure.pdf). In addition, participants (both funded and not) tend to view the process as exciting, engaging, and scientifically productive^2^ ^3^ .

Back in the USA, some similar observations were being made. A 2007 National Science Board report concluded that US research was sometimes stove-piped, and, in times of limited resources, reliable science projects are often preferred over risky but potentially transformative research 4 . While a lone investigator might successfully conduct risky and transformative research, the potential advantages of cross/multidisciplinary approaches were becoming more fully appreciated. Multidisciplinary teams could potentially bring novel perspectives and approaches to longstanding problems. In addition, scientists in other fields might not be vested in the current paradigm, so could ask the naïve questions that could lead to important new insights. More directly, in some cases a cognate problem in a related field may have already been approached or solved, so simply transferring that concept or approach to a new field could result in significant advances.

NSF staff felt that the UK term “Sandpit” (sandbox in the USA) did not translate well across the Atlantic, so settled on the phrase “Ideas Lab”. NSF’s first foray into Ideas Labs was a joint NSF/EPSRC effort in 2009 on Synthetic Biology 5 . This was followed by Ideas Labs on Innovations in Biological Imaging and Visualization (http://www.nsf.gov/publications/pub_summ.jsp?WT.z_pims_id=503473&ods_key=nsf10538) and Surpassing Evolution: Transformative Approaches to Enhance the Efficiency of Photosynthesis (http://www.nsf.gov/funding/pgm_summ.jsp?pims_id=503517, a joint event with the UK’s Biotechnology and Biological Sciences Research Council) in 2010, and AVAToL (http://www.nsf.gov/funding/pgm_summ.jsp?pims_id=503629) in 2011. An upcoming Ideas Lab is entitled Nitrogen: Improving on Nature (http://www.nsf.gov/publications/pub_summ.jsp?WT.z_pims_id=504773&ods_key=nsf12579). Many of the program officers involved with NSF Ideas Labs feel that they have been successful in generating ideas for risky and potentially transformative research of a type that may not have been generated nor funded through the standard review process.

The prior discussion gives some idea of the history and motivations for the Ideas Lab. What about the participants involved and the details of the process? The cast of players is large and diverse, with each group carrying out specific roles. These include:

- **Participants**: 20-30 participants from as broad a range of complementary relevant expertise as possible. The Ideas Lab process requires participants who are excited about ideas, who work well in a collaborative setting, and who are prepared to develop new ideas and directions (i.e., are not too set on a predetermined agenda). The Ideas Lab participants should be open-minded, flexible, genuinely curious people who are not too invested in their own ideas and are comfortable with the give-and-take of the process. Participants are the only players in the Ideas Lab process eligible for funding.

- **Mentors**: 3-5 mentors, who should also be from diverse backgrounds. The mentor role is complex, as mentors wear several hats throughout the process. Mentors assist in participant selection, connect and catalyze participants during the Ideas Lab process, provide feedback to the funders and facilitation team about idea development, and provide feedback to funders on developing ideas. The key question for mentors: Is the science exciting and novel?

- **Facilitators**: 3-4 in a team. Facilitators manage and drive the Ideas Lab process, but do not get involved in the content. NSF has used KnowInnovation (http://knowinnovation.com/), pioneers in the Ideas Lab/Sandpit process, as the facilitation team for its Ideas Labs.

- **Organizational Psychologist**: Provides guidance on participant selection based on applicants’ pre-proposals to ensure that selected participants will work collaboratively and productively in the intense Ideas Lab environment. The work of the organizational psychologist is subtle and critical; in general, the goal is to identify candidates who are open and flexible, enjoy working in groups, and do not come to the lab with a fixed idea or agenda (http://knowinnovation.com/brains-and-soul-in-equal-measure/). Program officers, who make the final decisions on participant selection, consider the guidance provided by the organizational psychologist and mentors.

- **Provocateurs**: Provocateurs are brought in to the Ideas Lab to challenge and stimulate the participants with brief, thought-provoking presentations.

- **Funders**: Provide funds. Funders receive feedback from mentors and facilitators on the process and make final funding decisions.

Once all of the players have been selected, how does the Ideas Lab proceed? First, a social networking site allows participants, mentors, and program officers to begin getting to know one another and exchanging ideas prior to the Ideas Lab.

A simplified explanation of the Ideas Lab meeting is as follows:

- Facilitators, through a series of activities, strive to stimulate creative thinking and problem solving, and to create an environment conducive to the free development and exchange of ideas.

- Participants, with continuous feedback from mentors, work to define the problem space. Within the overall problem space, what are the most important problems and obstacles to solving them?

- Participants familiarize themselves with the other participants and the skills they bring to problems within the purview of the Ideas Lab. What mix of participants has the interests and skills to attack a particular question?

- Groups of participants begin to coalesce around questions they find mutually interesting. Groups that have formed make presentations of ideas to other participants and mentors and are provided with constant, iterative feedback throughout the process from mentors and participants. Are the ideas interesting and potentially transformative? Groups are fluid, and may change membership over the course of the week as ideas are evaluated. Through repeated cycles of presentation and feedback, ideas are honed.

- At the end of the Ideas Lab, each group submits and presents a short proposal and rough budget. Mentors provide feedback to the funders on these proposals. Funders choose which groups will be invited to submit full proposals.

Subsequent to the Ideas Lab meeting, full proposals, due 2 months after the Ideas Lab, are evaluated for funding by NSF staff, with input from mentors. Following funding, groups get together for annual PI meetings to evaluate progress and continue to exchange ideas.

## NSF’s Assembling the Tree of Life Program and the Application of the Ideas Lab process to the Tree of Life (AVAToL)

The Assembling the Tree of Life (AToL) program was initiated by NSF in 2002, following substantial community input and several workshop reports (e.g., Systematics Agenda 2000, published in 1994 6 ). These workshop reports emphasized the compelling need and widespread benefit of determining the phylogenetic relationships among major groups of organisms, and of developing more powerful tools for phylogenetic analysis. The Tree of Life would facilitate comparative ecological and evolutionary studies, had the potential to integrate many areas of biology, and would accelerate our documentation and understanding of biodiversity.

NSF funded one round of Tree of Life grants through the Biocomplexity Program in 2001, and the first call for AToL proposals was published in FY2002. The program was originally managed (2002-2009) in the Biology Directorate as part of Emerging Frontiers, and then moved to the Division of Environmental Biology in 2010. To date, 48 projects have been funded including empirical phylogenetic projects covering large portions of the ToL ranging from viruses to vertebrates, as well as a wide range of phylogenetic methodological and computational projects. These investments have resulted in significant progress in understanding the origin and relationships of higher-level lineages such as major animal groups, land plants, fungi, protists, and new groups of Archeae; identifying the origins of keystone features of life such as multicellularity, bilateral symmetry, and the origins of mitochondria; discovery of evolutionary processes across the spectrum of the ToL including differential rates of genome evolution and horizontal gene transfer between lineages; and the adaptation and improvement of the sophisticated methods of phylogenomics, next generation sequencing, and bioinformatics for use in phylogenetics. We can now present a picture of the relationships among many of the deep branches of the Tree of Life, and considerable detail within some of these branches.

A new round of NSF community workshops funded by DEB’s Systematic Biology and Biodiversity Inventories program in 2009-10 7, as well as NSF’s internal assessment of the AToL program, identified several future challenges for the program:

- First, most progress to date in understanding the ToL had been within disjunct parts of the tree, using an investigator-driven, idiosyncratic approach for each independent AToL project. Disparate data were being collected and analyzed for each ToL project, with little or no coordination across ToL lineages. Therefore, data collected for inferring the ToL were heterogeneous and non-overlapping, presenting a challenge to integration. How can we integrate across the entire tree? Is whole genome data required? Is more centralized coordination the key? Should requirements exist for common core data sets for each project?

- Second, there are properties of the ToL that make it difficult to study: First, the ToL is enormous, including tens of millions of species. We are aware of only a fraction of these species, many of which are undocumented. Second, the ToL is very old, with a tremendous amount of phenotypic evolution having occurred along its branches. These properties have implications for how we might best prioritize research efforts. Missing lineages include woefully understudied groups (such as prokaryotes, many invertebrate groups, and most fossil lineages). How can we make progress in these areas? Should future ToL projects exclusively target understudied taxonomic groups? Should well-studied groups be excluded from the program for the present time? How can comparative morphology (whether derived from fossil or living taxa) be included in AToL projects, either in data matrices or otherwise?

- Third, the ToL is very complex and much of this complexity has been revealed through >10 years of large-scale AToL investments and research programs elsewhere in the world. For example, the tree of life is more complex than a simple tree: different genes can have different gene trees within a species; many organisms have multiple genomes that may have independent histories; other processes, such as lateral gene transfer, hybridization, and gene duplication or loss may cause dramatically differing gene trees, especially for certain groups. The ToL for prokaryotes is more controversial than that for eukaryotes as some of the confounding processes listed above are more frequent in prokaryotes by several orders of magnitude. How can the AToL program make progress in this area, as well as help clarify the role of these processes in evolutionary history across the ToL?

- Accelerating the pace of biodiversity documentation requires a ToL – but, ideally, such an acceleration will be achieved via a single, integrated, automated, updatable, dynamic ToL providing the phylogenetic infrastructure for all biodiversity 8. A universal, continually growing phylogenetic tree would provide the most powerful tool for biodiversity discovery, which is itself continuous and ongoing, and for efficient documentation of species and lineages. In concert with this, we need powerful visualization tools – tools that will enable visualization of ‘dark areas’ (poorly known or missing groups), identification of strongly supported vs. weakly supported areas of the tree, integration with all associated metadata (geographic, stratigraphic, vouchers), and navigation tools to traverse branches of the tree and processes occurring within the lineages.

Thus, 10 years after the AToL program began, and following major advances resulting from that investment, the community envisions the exciting prospect of inferring the ToL as an integrated, dynamic whole. NSF revised the AToL solicitation in FY2009 in order to solicit more synthetic proposals. The new solicitation identified the challenges listed above and also called attention to understudied taxonomic groups. The proposal budget cap (previously $3M per project) was lifted in order to accommodate potentially ambitious and transformative approaches. Specific goals that were emphasized included focusing on major understudied groups not yet addressed by current or previous AToL projects, as well as coordination across different AToL projects and identification of mechanisms to ensure better integration and compatibility across ToL projects. In response, some qualitatively different proposals were submitted and reviewed. However, it became apparent to program officers that ambitious proposals, particularly those seeking to change the field in new ways such as a more centralized approach to the ToL, would likely have difficulty within the traditional reviewing paradigm.

This is the very issue that has led to the Ideas Lab model: an interactive, residential workshop aimed at developing new and bold approaches to address grand challenges or problems that could benefit from a new dimension in thinking and that might be too risky, too much of a paradigm shift, or too unconventional to succeed within standard review practices. Around this same time, the NSF Biology Directorate began soliciting ideas from program officers for future Ideas Lab topics. The Systematic Biology program suggested “AVAToL – Assembling, Visualizing, and Analyzing the Tree of Life”. The main goals of AVAToL: identifying novel approaches for inferring the structure of the entire tree of life and its underlying data; developing innovative mechanisms for using our knowledge of the ToL to further our understanding of biological diversity and processes; and finding effective ways to visualize and communicate our knowledge to scientists, educators, and the public.

NSF’s AVAToL Ideas Lab solicitation was published in March 2011. The solicitation outlined the need to achieve integration and synthesis of separate phylogenies into a cohesive and dynamic tree for all of life, and to develop new tools for visualizing, querying, and further analyzing the ToL. Seventy-two preliminary proposals were received, which were reviewed by the five AVAToL mentors, an organizational psychologist, and NSF program officers. A diverse group of 25 systematists, biologists, paleontologists, computer scientists, and graphic designers were invited to participate in the intensive, five-day AVAToL Ideas Lab, which included project development and real-time peer review from other participants and a panel of reviewers. The fundamental goal of the activity was to identify opportunities for investment to significantly advance the state-of-the-art in tree construction, visualization, and analysis. The following two sections describe the process in more detail.

## The AVAToL Ideas Lab: Setting the Stage

After the AVAToL solicitation was published in March 2011, and the facilitators and organizational psychologist engaged, NSF program officers chose five mentors: two systematists who work primarily on recent taxa, a paleontologist, a computer scientist in phylogenetics, and a mathematician who works entirely outside the field of phylogenetics. Expertise of the mentors included evolutionary rates and the fossil record, evolution and development, phylogenetic methods and theory, software development, phyloinformatic databases, geometry, combinatorics, outreach, vertebrates and insects, morphological and genetic data, among others. Commitment of the mentors completed the team that provided administration and guidance prior to and during the Ideas Lab.

Preliminary proposals were submitted by individuals, not teams. Applicants came from fields close to the ToL, including phylogenetic biologists and computer scientists, as well as from more distant fields such as graphic arts and music. Each preliminary proposal contained information about the past experience of the individual, how they worked in collaborations, the reasons for their interest in AVAToL, and the research topics in which they would be interested.

These 72 preliminary proposals were then used to help choose whom to invite to the Ideas Lab. In contrast to typical NSF procedure, the proposals were used to judge which people would be appropriate for an Ideas Lab as much as which research ideas would be considered. With guidance from the facilitators and NSF, the mentors provided recommended rankings of participants to the program officers. With additional input from the organizational psychologist (who, based upon proposal content, evaluated potential participants based on their likely collaborative skills and success in creative groups), the NSF program officers then chose 25 people to invite. Overall, the goal was to choose participants with a diverse range of experiences and skills, and who were creative, community-minded, collaborative, and willing to go in novel directions. The participants included biologists representing most taxonomic domains, paleontologists, computer scientists, graphic artists, web designers, and science educators. A website was set up to allow participants to interact and exchange ideas before the Ideas Lab.

In addition to the participants, three provocateurs where chosen by the program officers after discussions with the mentors. Provocateurs included a science educator specializing in the teaching of evolution, a pure mathematician, and a phyloinformatician.

## The AVAToL Ideas Lab: As it Happened

The participants, mentors, program officers, provocateurs, and facilitators met in August 2011 at a quiet conference center in Lake Placid, New York, with all accommodation and meals provided in house. This created an environment that facilitated complete focus on the event at hand. The NSF program officers served solely as observers, and were available to answer questions as needed.

The four facilitators, experts in encouraging creativity and group dynamics in situations such as the Ideas Lab, orchestrated the gathering. It is hard to overemphasize how important these facilitators were to the process. The first four days of the meeting were designed by the facilitators to allow groups of participants to form naturally around compelling ideas, to increase the chances of success of each group, and to inspire creativity. Although in our experience academics are often skeptical about facilitators, the facilitators at the AVAToL Ideas Lab were so capable and well versed in running an Ideas Lab that the group quickly accepted and embraced the process. It was their friendly but firm presence that directed the entire meeting.

Early exercises were designed to first allow the participants to get to know each other and then to formulate a list of primary AVAToL challenges. For example, a large visual map of the domains of knowledge and problems in AVAToL was drawn by participants on a long wall; participants formed groups based upon their interest in particular “countries” on the map, with discussions about the exports each country might produce and their interactions with other countries. Later, a map of skills needed to tackle problems was created, with participants placing their names in regions in which they possessed relevant abilities; this later provided an excellent resource for groups as they sought particular expertise within the group for their nascent ideas. Participants were then asked to imagine a headline and article that might appear, after successful completion of an AVAToL project, in a newspaper or blog, announcing a major breakthrough. Groups coalesced around favored topics and presented a mockup of the front page of the newspaper or blog – an exercise that forced them to envisage their topic as a significant challenge, with major impacts beyond phylogenetics. Another exercise encouraged participants to categorize the major challenges and long-standing obstacles facing phylogenetics, including those that are often conveniently ignored (such as phenotypic/fossil data, and lateral gene transfer), and to brainstorm about how those obstacles might be tackled. Each exercise was designed to encourage participants to talk among themselves, to learn about each other’s strengths, style, and compatibility; to allow all to see the full breadth of the domain space; and to understand key challenges from multiple perspectives.

Many aspects of the meeting design encouraged interaction and creativity. For example, participants and mentors were regularly reassigned to different tables in the meeting rooms, encouraging acquaintances between previously unfamiliar people. This was especially important given the diversity of participants within biology, computer sciences, and in the arts and humanities, and given the diversity of skills needed for many of the AVAToL challenges.

The provocateurs each gave talks to challenge the group, forcing them to question their assumptions, step back to see the big picture, consider other aspects of society, or see things from the perspective of a different field.

As the week progressed, groups formed, each centered around a particular challenge that emerged from earlier exercises, then dispersed (by design), and reformed with potentially altered composition, and potentially altered topic. Group composition, and the targets of each group, began solidifying by mid-week, and the sessions became more focused. Multiple talks were given by each group, allowing them to pitch their challenge and proposal to all present, beginning with short talks about tentative proposals, progressing to more definitive talks. Anonymous feedback provided on post-it notes by other participants and mentors pointed out additional directions the teams may not have considered, enumerated remaining concerns, and allowed teams to gauge the level of enthusiasm for the proposed project. Later in the week, a mini-proposal was written by each group and submitted to the mentors for feedback. The process of preparing the talks and the Ideas-Lab-wide discussions that followed allowed each group to hone their objectives and their proposed methods to meet the challenge.

Even though the groups that eventually formed would be ultimately competing for funds, the atmosphere in the Ideas Lab was highly collegial and cooperative, with participants helping out other groups. This sense of community and shared purpose came in part from mutual passion among the participants to tackle the grand challenges of our field, and also from the skilled guidance of the mentors and facilitators.

Throughout this process, mentors were tasked with mingling among the groups, asking questions, challenging assumptions, connecting people, catalyzing (but not guiding) the conversations. Frequent meetings between the mentors and the facilitation team allowed the mentors to improve and fine-tune their interactions with the participants, and allowed time to solve any problems that arose.

On the last day of the Ideas Lab, each group gave a formal presentation of their proposed project, and then submitted a formal preproposal. That afternoon, preproposals were discussed by the mentors, who served as a panel giving recommendations about which projects should be invited to submit a full proposal. Within two weeks of the Ideas Lab meeting, three groups were invited by NSF to submit full proposals, due in two months. The mentors again served as a review panel, and gave recommendations to NSF about funding. In the end, three AVAToL projects were funded.

## The AVAToL Ideas Lab: Pros and Cons

From one mentor’s perspective [DM], the Ideas Lab was an impressive experience in group dynamics, and the ability of capable people to think big and envisage potential solutions to major research challenges, and to do this in only a few days’ time. The limited time and shared passions forced activation energies to be overcome quickly, diminished social concerns, added adrenaline, and solved problems with a clarity that can only be forged in the intensity of a deadline. By bringing together people who had never worked together before, and with very different skill sets, the process formed unexpected groups of people with abilities and perspectives lacking from many groups that form in the comfort zone of typical academic life.

From two program officers’ perspectives [TC, MK], the Ideas Lab was a remarkably different forum for the generation and review of research ideas. It is difficult to overstate the difference between observing the generation and fate of novel or risky ideas in a traditionally reviewed NSF proposal vs. within the context of the Ideas Lab. Willingness to entertain unconventional ideas and willingness to pursue the best directions for community progress vs. individual progress are two of the most obvious differences. The process also requires program officers to shed some of their own conservative tendencies and to be willing to risk a portion of their funding portfolio on possibly significant game-changers.

The nature of the Ideas Lab is not amenable to all participants nor all groups. Some participants might not naturally belong to any of the projects or find a home in groups that form during a Lab. Not joining a group means no funding and no reason to participate in the remainder of the Ideas Lab. This fact might compel everyone to join a group, even if it is a poor fit for them, and social norms make it difficult to reject such members from a group. In addition, a group that forms over four days might discover in the last couple of days that there are notable incompatibilities within the group, leading to a non-functional community, and a failed group and failed proposal, even if the group was working on a great idea. Another possibility is that stronger (especially more senior) personalities might dominate a group, and if, for example, they lead the group in a direction away from innovative, risky research, and toward more standard, ingrained research programs, then the group could fail to propose the necessarily risky science required for the challenges at hand. In the typically longer and socially less-intense course of formation of collaborations outside of an Ideas Lab, some of these group composition and dynamics issues are naturally resolved, but in the short window and close quarters of an Ideas Lab, that does not always happen.

At worst, individual groups within an Ideas Lab may fail to produce a full proposal that tackles a grand challenge in an appropriate way. This would result in no funds being awarded by NSF, and an unfortunate loss of an opportunity.

## The AVAToL Ideas Lab: Prospectus

Three AVAToL projects were funded in FY2012. One seeks to produce an online, comprehensive first-draft tree of all 1.8 million described species and to provide the phylogenetic infrastructure to facilitate a perpetually growing ToL thereafter. This “Open Tree of Life” effort includes development of many key improvements in phyloinformatics, with new algorithms for automatic updating of a synthetic ToL, tools to incentivize data deposition, and a community-driven approach to data contribution and data use (http://opentreeoflife.org). The second project (“Next Generation Phenomics”) seeks to bring phenotypic data into the next-generation revolution by adapting methods from computer vision, machine learning, and natural language processing to rapidly gather large phenotypic data sets across the entire ToL (http://phenomics.avatol.org). A third project, “Arbor” (http://www.arborworkflows.com), will build new evolutionary analysis workflows to allow one to visualize and analyze data on phylogenies at all scales, including the entire ToL.

Each project is of vast enough scale and tackles a complicated enough issue in phylogenetic biology that they, if successful, could transform the nature of phylogenetic research, biodiversity studies in general, and communication about life’s history. From OpenTree, a synthetic, automatically growing tree, incrementally extendible as new data arrives would be the core to a new, complete map of biodiversity. Such a map would allow much more rapid analysis and interpretation of new data. Molecular data will most easily be integrated into this framework, but critical phenotypic data cannot be easily gathered and incorporated without breakthrough methods, such as those being developed by the Next Generation Phenomics project. And the new analytical tools and pipelines from Arbor will integrate all of these data for novel, tree-of-life-scale analyses and visualizations. This will allow evolutionary processes (including those that complicate ToL studies and produce more web-like than tree-like structures) to be visualized and studied on a much more comprehensive scale.

Synthesizing a phylogeny of all the world’s species with DNA sequence and genomic data, with massive amounts of phenotypic data, and in a next-generation analysis and visualization pipeline, will dramatically improve research capabilities in evolutionary biology and biodiversity, and in all aspects of biology that compare data from multiple species or individuals. It will also provide an unprecedented platform to communicate to the public about the history of life and the origins of all of the forms, behaviors, molecules, and interactions of life on earth.

The goals of AVAToL are grand, and the projects are risky; they are so bold as to have a higher likelihood of failure than standard collaborative projects. But the challenges in phylogenetics are of a scale that we cannot expect the long-standing roadblocks to be overcome with standard science.
